# Setting the stage for novel public policy and fiscal impact studies regarding the economics of in vitro fertilisation: An introduction

**Published:** 2010-01-15

**Authors:** David J. Walsh, Anthony P.H. Walsh

**Affiliations:** The Sims Institute/Sims International Fertility Clinc; Dublin, Ireland

## Background

Effective health policy requires a thorough understanding of intrinsic social, ethical, political and philosophical aspects of infertility and its treatment. The procedure with the highest success rate to address the clinical challenge of infertility for many patients is a relatively high-technology procedure, in vitro fertilisation (IVF).

## Introduction

More than half of all reported IVF cycles undertaken worldwide occur in Europe [1], yet there is considerable diversity in European public funding strategies for treating infertility [2]. Prevailing reimbursement guidelines have been developed to assuage safety and regulatory concerns, although economic factors are also important. In the context of public policy debate, arguments to restrict public funding for IVF are generally supported by the observation that treatment should be preferentially provided to younger women [3] because published data has shown that the effectiveness of IVF declines with female age >40yrs [4,5]. This stance seeks to exclude older women who are more likely to have a greater need for IVF [2]. Policy decisions involving the growth of family are important; any eligibility criteria must be supported by studies without methodological limitations in order to capture the broadest possible cost/benefit analysis of IVF. It has recently been recognised that the available fiscal data on the economics of IVF is indeed limited, and should be augmented by more robust evidence to guide policy makers in the development of particular social reimbursement schemes [2]. Accordingly, this paper introduces the following body of new research based on a comprehensive investigation on the monetary, regulatory and policy factors that drive the cost of IVF.

In subsequent issues, The *Journal of Experimental & Clinical Assisted Reproduction* (JECAR) presents results from detailed investigations on IVF gathered in the U.K. by Dr. Christopher Jones and colleagues. These studies represent definitive evaluations of the cost-effectiveness of IVF, designed for a wide readership— practitioners, medical consumers and policy makers. With an emphasis on various embryo transfer policies, the authors present IVF protocols grouped by cost-effectiveness for specified clinical populations. Against a background of clinical IVF practice in the U.K. National Health Service (NHS), definitions and descriptions of all relevant terms are provided. The authors also provide a computational analysis of a generic IVF cycle, including a synopsis of key personnel involved in daily clinic operation. This enables an objective calculation of relative contributions of manpower and supplies consumed in a typical IVF cycle. Based on U.K. data derived from the Human Fertilisation & Embryology Authority (HFEA), an incremental cost-effectiveness analysis estimates extra costs for an additional livebirth event, showing how additional embryos transferred may be cost-effective for certain patient populations.

Recognising the significance of multiple gestation following IVF, this team explores health care cost models based on data from neonatal intensive care and special care baby units; length of stay (LOS) as a function of plurality and gestational age at delivery is also investigated. Few previous studies account for these indirect costs of IVF, and therefore may result in an underestimation of the total socioeconomic impact of IVF [6]. Jones and colleagues relate LOS across male vs. female singletons, twins and triplets. The economics of rare reproductive outcomes, including multiple birth following single embryo transfer, are also considered.

## Conclusion

From civilisation’s earliest beginnings, the stigma of infertility has been a recognised cultural phenomenon. How best to use limited health care resources to confront this challenge in modern times remains controversial, in part due to a paucity of economic data [7]. IVF is now regarded as a safe and effective remedy to infertility for many couples [8]. Providing a fresh perspective on a familiar problem, the forthcoming papers by Jones and colleagues address this difficult issue from the U.K. perspective. The recognised need for a thorough and robust economic assessment of IVF is, in large measure, addressed by the investigative efforts of Jones and colleagues. In developing their cost/benefit analysis of such a heavily-regulated clinical intervention like IVF in the U.K., the authors have acknowledged that the need to focus on individual patients and/or providers is eclipsed by the need to deal with the underlying policy frameworks that guide them. Evidence is provided to show how U.K. government policy has already been modified in response to these data. It is hoped that further studies in other jurisdictions will lead to better informed policy debates elsewhere, and JECAR welcomes an opportunity to consider those reports for potential publication when available.
